# Astrocytes and Microglia as Potential Contributors to the Pathogenesis of *C9orf72* Repeat Expansion-Associated FTLD and ALS

**DOI:** 10.3389/fnins.2019.00486

**Published:** 2019-05-15

**Authors:** Hannah Rostalski, Stina Leskelä, Nadine Huber, Kasper Katisko, Antti Cajanus, Eino Solje, Mikael Marttinen, Teemu Natunen, Anne M. Remes, Mikko Hiltunen, Annakaisa Haapasalo

**Affiliations:** ^1^A.I. Virtanen Institute for Molecular Sciences, University of Eastern Finland, Kuopio, Finland; ^2^Institute of Clinical Medicine – Neurology, University of Eastern Finland, Kuopio, Finland; ^3^Neuro Center, Neurology, Kuopio University Hospital, Kuopio, Finland; ^4^Institute of Biomedicine, University of Eastern Finland, Kuopio, Finland; ^5^Medical Research Center, Oulu University Hospital, Oulu, Finland; ^6^Research Unit of Clinical Neuroscience, Neurology, University of Oulu, Oulu, Finland

**Keywords:** amyotrophic lateral sclerosis, astrocyte, *C9orf72*, *C9orf72* expansion, frontotemporal lobar degeneration, microglia, neurodegeneration

## Abstract

Frontotemporal lobar degeneration (FTLD) and amyotrophic lateral sclerosis (ALS) are neurodegenerative diseases with a complex, but often overlapping, genetic and pathobiological background and thus they are considered to form a disease spectrum. Although neurons are the principal cells affected in FTLD and ALS, increasing amount of evidence has recently proposed that other central nervous system-resident cells, including microglia and astrocytes, may also play roles in neurodegeneration in these diseases. Therefore, deciphering the mechanisms underlying the disease pathogenesis in different types of brain cells is fundamental in order to understand the etiology of these disorders. The major genetic cause of FTLD and ALS is a hexanucleotide repeat expansion (HRE) in the intronic region of the *C9orf72* gene. In neurons, specific pathological hallmarks, including decreased expression of the *C9orf72* RNA and proteins and generation of toxic RNA and protein species, and their downstream effects have been linked to *C9orf72* HRE-associated FTLD and ALS. In contrast, it is still poorly known to which extent these pathological changes are presented in other brain cells. Here, we summarize the current literature on the potential role of astrocytes and microglia in *C9orf72* HRE-linked FTLD and ALS and discuss their possible phenotypic alterations and neurotoxic mechanisms that may contribute to neurodegeneration in these diseases.

## Introduction

Frontotemporal lobar degeneration (FTLD) is a group of neurodegenerative disorders affecting predominantly the frontal and temporal lobes of the brain (Gorno-Tempini et al., 2011; Rascovsky et al., 2011) and the second most prevalent early-onset dementia (Onyike and Diehl-Schmid, 2013). FTLD clinical syndromes are characterized by changes in behavior, personality, and executive functions or deterioration of language functions. *GGGGCC* hexanucleotide repeat expansion (HRE) in the non-coding region of *chromosome 9 open reading frame 72* (*C9orf72*) (C9-HRE) is the major genetic cause of familial FTLD (12–48%) and amyotrophic lateral sclerosis (ALS) (24–46%) cases and 6–20% of sporadic cases for both diseases (DeJesus-Hernandez et al., 2011; Renton et al., 2011; Majounie et al., 2012). C9-HRE may also lead to concomitant FTLD and ALS. The pathobiology of C9-HRE-associated FTLD and ALS (C9-FTLD or C9-ALS) is complex. Neurons in the C9-HRE carriers display specific pathological hallmarks, including toxic RNA and proteins derived from the expanded C9-HRE and decreased expression of *C9orf72* due to haploinsufficiency (see reviews Gitler and Tsuiji, 2016; Freibaum and Taylor, 2017; Balendra and Isaacs, 2018). However, recently the potential role of other central nervous system (CNS)-resident cells, especially astrocytes and microglia, has also started to gain attention.

Glial cells are essential for brain homeostasis, but they also may mediate neuroinflammation (Jang et al., 2013; Franco and Fernández-Suárez, 2015; Shinozaki et al., 2017). Chronic changes in their physiological functions may contribute to neurodegeneration via both cell autonomous and non-cell autonomous mechanisms in neurodegenerative diseases, including ALS and FTLD. Whereas, most of the early findings on glial involvement in ALS pathogenesis derived from studies on mutant superoxide dismutase 1 (SOD1), there is accumulating evidence for glial contribution in other subtypes of ALS as well (Broe et al., 2004; Haidet-Phillips et al., 2011; Minami et al., 2015; Radford et al., 2015; Chen et al., 2016; Lee et al., 2016; Taylor et al., 2016; Cooper-Knock et al., 2017; Hallmann et al., 2017; Krabbe et al., 2017; Bachiller et al., 2018; Deczkowska et al., 2018); for a recent review on the role of neuroinflammation and complement system in ALS see also (Parker et al., 2019). Communication between neurons and glia via secreted factors and membrane-bound receptors is crucial for e.g., regulation of synaptic pruning and detection and clearance of apoptotic cells, and alterations in this crosstalk are suggested to contribute to the pathogenesis of neurodegenerative diseases (Rama Rao and Kielian, 2015; Szepesi et al., 2018). For example, complement 3 (C3) levels are increased, whereas the levels of signal regulatory protein (SIRP) α, a protein negatively regulating phagocytosis, and its corresponding receptor on microglia, cluster of differentiation (CD) 47, are reduced in the frontal cortex of FTLD patients compared to healthy controls or ALS and FTLD/ALS patients (Gitik et al., 2011; Umoh et al., 2018). Also, levels of CD200, expressed on neurons and restricting microglial activation (Barclay et al., 2002), are reduced in the frontal cortex of FTLD compared to FTLD/ALS patients (Umoh et al., 2018). These findings imply that microglia-mediated synaptic pruning and phagocytosis might be enhanced in FTLD brains. RNA expression analyses have indicated that pathways including the complement system, antigen presentation, and interferon (IFN) γ and interleukin (IL) 1-β signaling are significantly upregulated in the brains of C9-ALS patients compared to sporadic ALS patients (Prudencio et al., 2015; O'Rourke et al., 2016). Also transcription factors of the nuclear factor kappa B (NFκB) pathway were differentially expressed in C9-ALS and non-C9-ALS patients compared to healthy subjects (Ismail et al., 2013), but it is unclear in which cells these alterations occur. Lower levels of C-X-C motif chemokine ligand 10 protein, a microglial chemoattractant, in the cerebrospinal fluid (CSF) of C9-ALS patients were observed compared to non-C9-ALS cases (Ismail et al., 2013), but the physiological consequences of this are unknown. Astrocytes from C9-ALS patients show glucose hypermetabolism as compared to non-C9-ALS cases and controls, possibly as a consequence of astrogliosis (Cistaro et al., 2014). Decreased levels of excitatory amino acid transporter (EAAT) 2 in the frontal cortex of FTLD patients compared to controls (Umoh et al., 2018) and in C9-ALS compared to sporadic ALS patients (Fomin et al., 2018) suggest that astrocytes in both C9-HRE carriers and non-carriers might show defective uptake of glutamate, which could lead to excitotoxicity. Thus, mounting evidence points to a potentially altered physiology of microglia and astrocytes in FTLD and ALS associated with C9-HRE.

## Pathological Hallmarks of C9-HRE in Glial Cells

### RNA Foci Are Less Abundant in Glial Cells Compared to Neurons

Sense and antisense RNA foci, formed by aggregated C9-HRE-containing RNA, represent a unique pathological feature of C9-FTLD and C9-ALS. RNA foci have mainly been reported in neurons (DeJesus-Hernandez et al., 2011; Renton et al., 2011; Gendron et al., 2013; Mizielinska et al., 2013; Dafinca et al., 2016), but also in non-neuronal cells, such as fibroblasts and lymphoblasts (Donnelly et al., 2013; Lagier-Tourenne et al., 2013; Cooper-Knock et al., 2014). Interestingly, glial fibrillary acidic protein (GFAP)-positive and negative glial cells in the cerebellum of C9-ALS and C9-FTLD cases (Gendron et al., 2013) and induced pluripotent stem cell (iPSC)-derived astroglia have also been confirmed to contain sense foci (Sareen et al., 2013). However, compared to neurons, RNA foci have only been detected in a small fraction of microglia and astrocytes in *post-mortem* C9-ALS frontal cortex (Mizielinska et al., 2013) and/or cerebellum of C9-HRE carrying FTLD, ALS and FTLD/ALS patients (DeJesus-Hernandez et al., 2017). Furthermore, the number of RNA foci per cell was lower in microglia and astrocytes compared to neurons (Mizielinska et al., 2013). Whereas, neurons may exhibit nuclear and, to a lower extent, cytoplasmic RNA foci, microglia, and astrocytes showed only intranuclear RNA foci (Lagier-Tourenne et al., 2013; Mizielinska et al., 2013). This may suggest that (i) glial cells might not express C9-HRE to the same extent as neurons; (ii) expression of RNA-binding proteins, known to stabilize RNA foci, and/or proteins involved in cytoplasmic translocation of C9-HRE RNA are less abundant; (iii) glial cells can better clear C9-HRE-containing RNA (Peters et al., 2015); or (iv) somatic heterogeneity of the C9-HRE length, which can occur in different cells within the same tissue of C9-HRE carriers (DeJesus-Hernandez et al., 2011; Almeida et al., 2013; van Blitterswijk et al., 2013; Nordin et al., 2015), might underlie the lower prevalence of RNA foci in glial cells compared to neurons. The RNA foci in neurons are suggested to cause disturbances in RNA metabolism through sequestration of RNA-binding proteins (Donnelly et al., 2013; Mori et al., 2013a; Sareen et al., 2013; Cooper-Knock et al., 2014). Similar effects might occur in glial cells exhibiting RNA foci, but further investigations are required to ascertain this.

### Dipeptide Repeat Proteins Appear Less Frequent in Glial Cells Than Neurons

**Figure 1:**
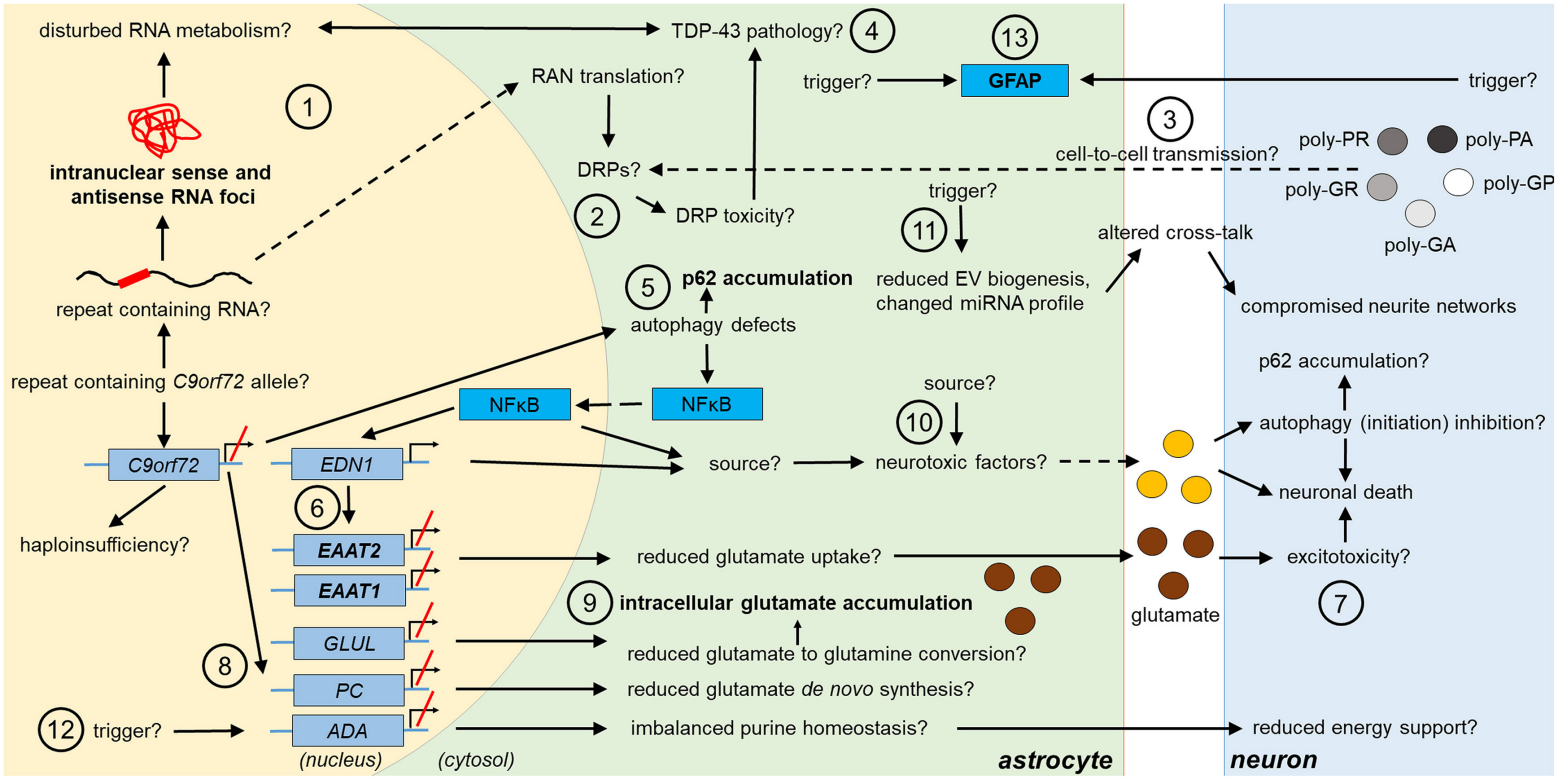
Potential and confirmed phenotypic features of *C9orf72* HRE*-*associated astrocytes in FTLD/ALS. Features detected in the astrocytes of FTLD or ALS patient *post-mortem* brain are indicated in bold text. The presence of the typical C9-HRE-associated pathological hallmarks, which have previously been observed mainly in neurons, as well as other potential mechanisms, which still need to be confirmed in human patient astrocytes, are indicated with a question mark. Directions of sequential events are visualized with arrows. Steps requiring intracellular or intercellular translocation of molecules are indicated by dashed arrows. The different events are indicated by numbers as follows: **(1)** C9-HRE-containing RNA might be transcribed in astrocytes, forming intranuclear sense and antisense RNA foci, which may disturb RNA metabolism. **(2)** C9-HRE-containing RNA might be translocated into the cytosol of astrocytes, where it could undergo RAN translation creating potentially toxic DRPs. **(3)** DRPs might also be transmitted from other cell types, such as neurons, to astrocytes. **(4)** Potential disturbances in RNA metabolism as well as DRP toxicity might lead to TDP-43 pathology, which in turn could lead to defects in RNA processing in astrocytes. **(5)** Reduced C9orf72 levels in astrocytes might cause defects in autophagy, resulting in p62 accumulation and increase of NFκB levels in the cytosol and nucleus. **(6)** Enhanced NFκB levels with simultaneously decreased C9orf72 levels might enhance *EDN1* expression, which suppresses *EAAT1* and 2 expression in astrocytes. **(7)** As a result, astrocytic uptake of extracellular glutamate might be diminished, which might lead to excitotoxicity. **(8)** Reduced *C9orf72* levels might lead to decreased expression of genes involved in glutamate *de novo* synthesis (e.g., *PC*) and glutamate to glutamine conversion (e.g., *GLUL*). **(9)** Reduced conversion of glutamate to glutamine might underlie intracellular glutamate accumulation. **(10)** Neurotoxic factors, which partly might be created through the altered NFκB signaling, cause neuronal death. Neurotoxic factors might cause autophagy inhibition in neurons, which might lead to p62 accumulation. **(11)** Reduced EV synthesis, which might lead to decreased EV secretion, and an altered miRNA expression profile, might influence the crosstalk between astrocytes and neurons, which could lead to disturbed neurite growth and networks. **(12)** Decreased expression of *ADA* might disrupt purine metabolism and lead to decreased energy support of neurons by astrocytes. **(13)** Enhanced GFAP expression might derive from endogenous or exogenous triggers. ADA, adenosine deaminase; DRP, dipeptide repeat protein; EAAT1/2, excitatory amino acid transporter1/2; EDN1, endothelin 1; EV, extracellular vesicle; GFAP, glial fibrillary acidic protein; GLUL, glutamate-ammonia ligase; HRE, hexanucleotide repeat expansion; miRNA, micro ribonucleic acid; NFκB, nuclear factor “kappa-light-chain-enhancer” of activated B-cells; PC, pyruvate carboxylase; RAN, repeat-associated non-AUG; TDP-43, Transactive response DNA-binding protein 43.

**Figure 2:**
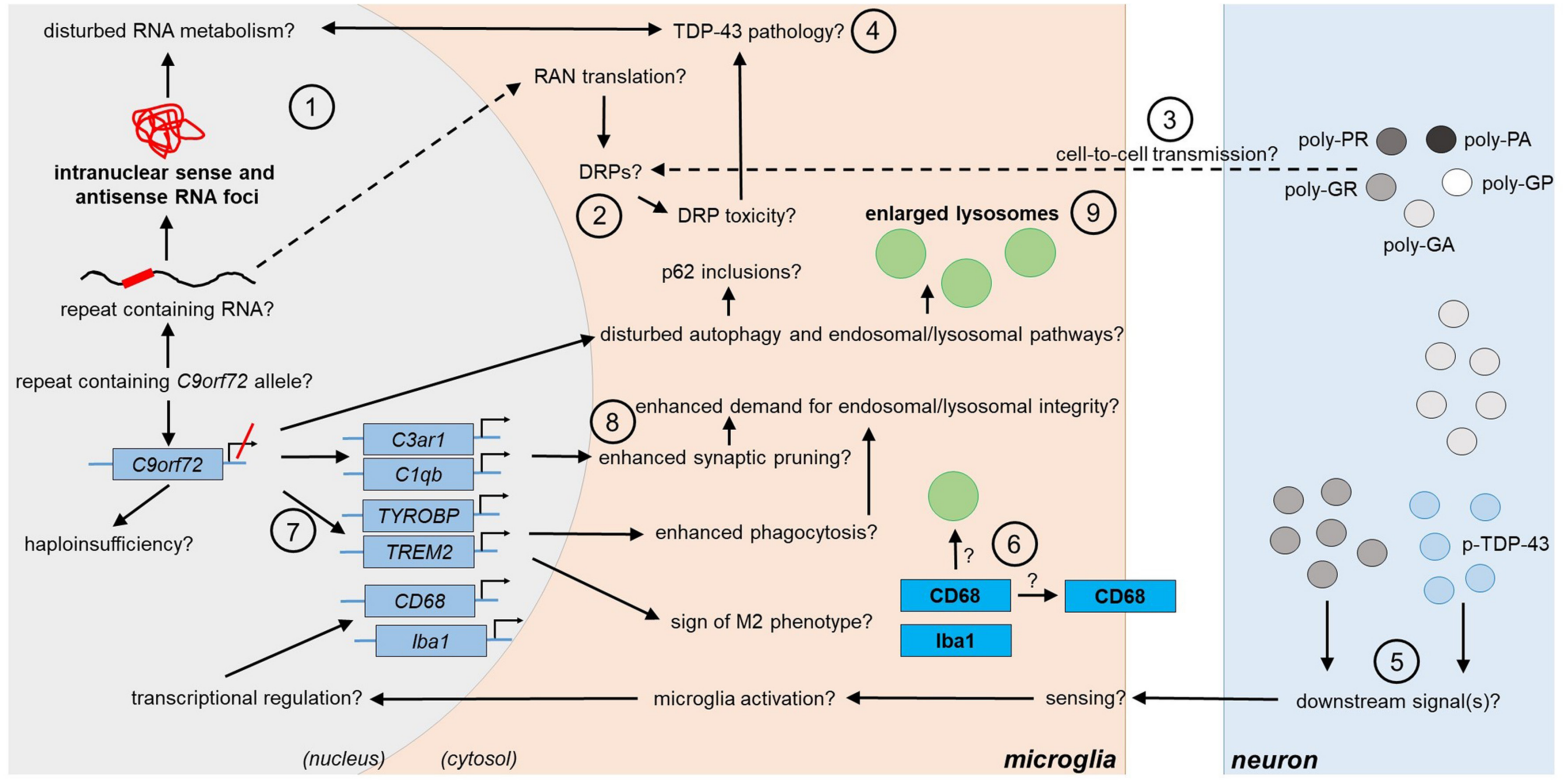
Potential and confirmed phenotypic features of *C9orf72* HRE-associated microglia in FTLD/ALS. Features detected in the microglia of FTLD or ALS patient *post-mortem* brain are indicated in bold text. The presence of the typical C9-HRE-associated pathological hallmarks, which have previously been observed mainly in neurons, as well as other potential mechanisms, which still need to be confirmed in human patient microglia, are indicated with a question mark. Directions of sequential events are visualized with arrows. Steps requiring intracellular or intercellular translocation of molecules are indicated by dashed arrows. The different events are indicated by numbers as follows: **(1)** C9-HRE-containing RNA might be transcribed in microglia, forming intranuclear sense and antisense RNA foci, which may disturb RNA metabolism. **(2)** C9-HRE-containing RNA might be translocated into the cytosol of microglia, where it could undergo RAN translation creating potentially toxic DRPs. **(3)** DRPs might also be transmitted from other cell types, such as neurons, to microglia. **(4)** Potential disturbances in RNA metabolism as well as DRP toxicity might lead to TDP-43 pathology, which in turn could lead to defects in RNA processing in microglia. **(5)** Expression of poly-GA in combination with enhanced TDP-43 phosphorylation (p-TDP-43) or poly-GR expression alone in neurons might lead to downstream signals, which are recognized by adjacent microglia, leading first to enhanced *CD68* and later *Iba1* expression. **(6)** CD68 could serve as a receptor on the cell surface or localize in lysosomes. **(7)** Downregulation of *C9orf72* might increase *TREM2* and *TYROBP* expression, which might be a sign of M2 microglial phenotype switching and result in increased phagocytic activity. **(8)** Microglial *C3ar1* and *C1qb* expression might be increased through decreased expression of *C9orf72* and lead to enhanced synaptic pruning. **(9)** Decreased *C9orf72* expression might disturb autophagy pathway, resulting in p62 accumulation and enlarged lysosomes in microglia. C1qb, complement subcomponent C1q chain B; C3ar1, complement C3a Receptor 1; CD68, cluster of differentiation 68; DRP, dipeptide repeat protein; HRE, hexanucleotide repeat expansion; Iba1, ionized calcium-binding adapter molecule 1; RAN, repeat-associated non-AUG; TDP-43, Transactive response DNA binding protein 43; TREM2, Triggering receptor expressed on myeloid cells 2; TYROBP, tyrosine kinase binding protein.

In addition to the RNA foci, five dipeptide repeat protein (DRP) species, namely poly-glycine-alanine (poly-GA), poly-glycine-proline (poly-GP), poly-glycine-arginine (poly-GR), poly-proline-arginine (poly-PR), and poly-proline-alanine (poly-PA) are directly derived via repeat-associated non-AUG (RAN) translation from the C9-HRE-containing RNA and represent additional pathological hallmarks unique to C9-FTLD and C9-ALS (Zu et al., 2011, 2013; Ash et al., 2013; Gendron et al., 2013; Mori et al., 2013b). Consistent with the low prevalence of RNA foci in glia, poly-GA inclusions were not detected in microglia or astrocytes of *post-mortem* C9-FTLD or C9-ALS brains (Mackenzie et al., 2013) or in astrocytes in the hippocampus of a C9-ALS patient (Ash et al., 2013). Also, poly-GP inclusions were undetectable in glial cells of C9-ALS and C9-FTLD patients showing sense RNA foci in cerebellar astrocytes (Gendron et al., 2013). Another study on C9-ALS *post-mortem* brains could not detect any of the DRPs in glial cells in subcortical white matter, hippocampus or white matter in the spinal cord, where glial cells are abundant (Saberi et al., 2018). However, poly-GA, poly-GP and poly-GR inclusions were detected in the ependymal cells of the spinal cord central canal of C9-FTLD and C9-FTLD/ALS cases. Poly-GA inclusions were also observed in ependymal and subependymal cells of the lateral ventricular wall (Schludi et al., 2015). These results suggest that DRP inclusions might be present in glial cells, but to a lower extent than in neurons and in defined CNS areas. Use of cell type-specific antibodies may help to decipher which glial cell types exhibit DRP inclusions and whether they, if present, compromise glial cell function. The lower prevalence of DRPs in glia as compared to neurons could mean that (i) less C9-HRE-containing RNA is translocated into the cytosol; ii) it undergoes RAN translation less efficiently; (iii) and/or DRPs are degraded before they can accumulate. These might be supported by the finding that adeno-associated virus (AAV)-mediated expression of DRPs in mice leads to DRP accumulation in neurons but not glial cells (Chew et al., 2015). However, it cannot be excluded that glial DRPs might in fact derive from DRPs secreted by neurons, since neuron-to-glia transmission has been shown to occur *in vitro* (Westergard et al., 2016) (Figures 1, 2).

### Glial Cells of C9-ALS and C9-FTLD Brain Present TDP-43 Pathology and p62 Inclusions

Transactive response DNA binding protein 43 (TDP-43) phosphorylation, cytoplasmic translocation, and truncation are pathological hallmarks of FTLD and ALS, including C9*-*FTLD and C9-ALS (Cooper-Knock et al., 2012), and potential contributors to disturbed RNA metabolism (Gendron et al., 2010). Neuronal TDP-43-negative, but p62-positive inclusions containing DRPs, represent another unique hallmark of C9-HRE (Mahoney et al., 2012). It has been suggested that TDP-43 aggregation can be caused by *C9orf72* haploinsufficiency, formation of RNA foci, or DRP toxicity (Cooper-Knock et al., 2015; Sellier et al., 2016; Nonaka et al., 2018), whereas p62 accumulation might result from *C9orf72* haploinsufficiency via impairment of autophagy (Sellier et al., 2016) or DRP expression (May et al., 2014). Cytoplasmic p62 and (phospho)-TDP-43-positive inclusions have been reported in glia in frontal, parietal, temporal, and motor cortex, hippocampus, brainstem, cerebellum, and spinal cord of C9-FTLD, C9-FTLD/ALS, and C9-ALS cases (Al-Sarraj et al., 2011; Cooper-Knock et al., 2012; Schipper et al., 2016), but it was not specified in which glial cell types the inclusions were detected. Some studies have reported phospho-TDP-43-positive inclusions in oligodendrocytes (Murray et al., 2011; Brettschneider et al., 2014; Fatima et al., 2015) and p62-positive inclusions in astrocytes (Simón-Sánchez et al., 2012). Co-immunostaining with cell type-specific antibodies would provide clarification to which extent astrocytes and microglia display p62 and TDP-43 inclusions. Understanding how the accumulation and aggregation of these proteins affect glial cell function could yield mechanistic insights into their potential contribution to disease pathogenesis. In conclusion, so far C9-HRE-associated pathological hallmarks have been detected to a lower extent in glial cells than in neurons in human *post-mortem* brains. In addition to the abovementioned potential reasons underlying the lower prevalence of these hallmarks in glial cells, yet one other option might be that glial cells can undergo extensive proliferation (Michell-Robinson et al., 2015; Verkhratsky and Nedergaard, 2018), which could either prevent the formation or dilute the amount of already existing RNA foci, DRPs, protein aggregates, or inclusions.

## Astrocytes in Model Systems of C9-FTLD and C9-ALS

### C9-HRE Might Cause Astrogliosis

Increased chitinase-3-like protein 1 (CHI3L1) and GFAP expression are considered as indicators of astrogliosis (Sofroniew and Vinters, 2010; Zamanian et al., 2012), and both proteins show increased levels in the frontal cortex and CSF of FTLD patients (Umoh et al., 2018; Oeckl et al., 2019). Interestingly, elevated GFAP expression was detected in mice upon AAV-mediated C9-HRE expression and in one bacterial artificial chromosome (BAC) C9-HRE mouse model (Liu et al., 2016). However, in two other BAC mice, signs of astrogliosis or microgliosis were not detected (Peters et al., 2015; Jiang et al., 2016). It should be noted that these mice did not concomitantly model haploinsufficiency. Astrocytes of *C9orf72*^−/−^ mice did not show enhanced GFAP immunoreactivity, indicating that lack of *C9orf72* does not cause astrogliosis (Koppers et al., 2015). Increased GFAP levels do not always correlate with enhanced ionized calcium-binding adapter molecule (Iba)1 levels (Zhang et al., 2016; Schludi et al., 2017), suggesting that trigger(s) for astro- and microgliosis are different. Identification of such triggers might be essential for elucidating pathogenic disease mechanisms.

### Neurotoxicity Mediated by Astrocytes Containing C9-HRE

Recent studies suggest that astrocytes from C9-HRE carriers with ALS can mediate neurotoxicity. Murine embryonic stem cell-derived motor neurons co-cultured with fibroblast-derived astrocytes from sporadic ALS or C9-ALS patients underwent extensive cell death within 4 days. Partial replacement of the culture medium by control astrocyte conditioned medium (ACM) did not prevent cell death, suggesting involvement of a gain-of-toxic-function mechanism rather than insufficient trophic support by the C9-HRE astrocytes (Meyer et al., 2014). Moreover, iPSC-derived motor neurons from control subjects or C9-ALS patients cultured in C9-ALS ACM showed dramatically decreased viability after 5 days (Madill et al., 2017). These studies indicate that direct physical contact between C9-HRE astrocytes and neurons as well as secretion of neurotoxicants might mediate neuronal cell death in a non-cell autonomous manner. Varcianna et al. showed that extracellular vesicles (EVs) secreted by induced astrocytes from C9-ALS patients decreased motor neuron survival. Also, C9-HRE astrocytes revealed a profile of increased or decreased expression of certain microRNAs compared to healthy controls, of which many are involved in axonal guidance and maintenance. Among these, miRNA-494-3p, which was recently shown to protect against lipopolysaccharide-induced cell death by targeting IL-13 expression (Geng and Liu, 2019), was significantly reduced in EVs secreted by C9-HRE astrocytes. Treatment with miRNA-494-3p mimic restored neurite length and number of nodes, and promoted motor neuron survival, suggesting that C9-HRE astrocytes might have impaired capacity to support neurons. This lack of support might also partially involve impaired EV biogenesis in astrocytes, which has also been shown to take place in fibroblasts and motor neurons derived from C9-HRE carriers (Aoki et al., 2017; Varcianna et al., 2019). Addition of the ACM from the same C9-HRE astrocytes led to slightly decreased cell survival compared to EVs only, indicating that in addition to EVs, also other astrocyte-derived soluble factors might cause neurotoxicity (Varcianna et al., 2019). In addition, impaired autophagy initiation and increased levels of SOD1 in neurons have been proposed as possible underlying mechanisms of the neurotoxicity mediated by C9-HRE-containing astrocytes through secreted factors (Madill et al., 2017), but require further investigations. Since SOD1 is regulated by transcription factors, such as NFκB (Milani et al., 2011), which in turn are controlled by environmental stimuli, investigating the mechanism behind increased SOD1 levels might yield better understanding on mechanisms of astrocyte-mediated neurotoxicity. Since autophagy can be regulated via microRNAs (Shah et al., 2018), investigating whether microRNAs secreted by C9-HRE astrocytes are the underlying mechanism of the inhibition of autophagy initiation in co-cultured cells would be interesting in order to find therapeutic targets.

Notably, under stress conditions, such as increased extracellular adenosine levels, neurotoxicity might be even enhanced. This idea is supported by the finding that induced sporadic as well as C9-HRE astrocytes and neurons harbor lower levels of adenosine deaminase (ADA), which normally deaminates adenosine to inosine. This predisposition has been shown to lead to enhanced death of the induced astrocytes themselves as well as motor neurons when cultured together with C9-HRE or sporadic induced astrocytes. Several conditions can lead to elevated adenosine triphosphate (ATP) levels in the brain and these can be sensed by and activate microglia and astrocytes. Under normal conditions, excessive ATP can be metabolized. However, defective ATP metabolism, as e.g., during ADA deficiency, could lead to excessive glial activation and subsequent neuroinflammation. In addition, decreased ADA levels could disturb energy metabolism in astrocytes and result in impaired nutritional support for neurons by C9-HRE astrocytes (Allen et al., 2019).

### Haploinsufficiency as a Potential Mechanism of Astrocyte-Mediated Neurotoxicity

In humans, two C9orf72 protein isoforms, long (~50 kDa) and short (~25 kDa), have been described. The levels of both protein isoforms are reduced in C9-HRE carrier tissues, including brain areas affected by neurodegeneration (Saberi et al., 2018). siRNA-mediated knockdown of both C9orf72 isoforms in U87 glioblastoma cells or normal human astrocytes has been shown to lead to the accumulation of p62 inclusions (Fomin et al., 2018), supporting the idea that loss of *C9orf72* may lead to their formation. Also, reduced expression of *pyruvate carboxylase* (*PC*), *EAAT1* and *2*, and *glutamine synthetase* (*GLUL*) together with intracellular glutamate accumulation was observed, implying that disturbed glutamate *de novo* synthesis, uptake and conversion into glutamine, all crucial functions in astrocytes, may take place upon *C9orf72* knockdown (Fomin et al., 2018). Decreased *EAAT1* and *EAAT2* levels suggest that C9-HRE astrocytes may not be able to efficiently take up excessive glutamate from synaptic cleft, which might lead to glutamate excitotoxicity, especially as induced motor neurons of C9-HRE ALS patients and *C9orf72*-deficient motor neurons have higher levels of glutamate receptors in neurites and dendritic spines (Shi et al., 2018). It was also observed that expression of *endothelin* (*EDN*) *1* as well as the levels of cytosolic and nuclear NFκB p65 were increased. The authors showed that the short C9orf72 isoform can bind to the predicted NFκB promoter binding site in *EDN1* and further suggested that in *C9orf72* knockdown cells, increased NFκB expression could lead to increased expression of *EDN1*, a negative regulator of *EAAT2* expression (Fomin et al., 2018). Therefore, the mechanisms underlying C9-HRE astrocyte-mediated neurotoxicity might involve *C9orf72* haploinsufficiency and p62 accumulation, and enhanced *EDN1* expression and NFκB signaling, known to induce the expression of nitric oxide synthase (Fomin et al., 2018).

Knockdown of both C9orf72 isoforms in U251 human astroglioma cells increased transmembrane protein 106b (TMEM106b) and progranulin but not lysosomal-associated membrane protein (LAMP) 1 and cathepsin D protein levels (similar to microglia in *C9orf72-*deficient mice Sullivan et al., 2016, see below). Similar effect was not detected when the cells were only expressing C9-HRE (Nicholson et al., 2018). These findings implicate that *C9orf72* haploinsufficiency may cause changes in astrocytic lysosomal pathways. Whether such effects can be observed in C9-HRE carriers and how they might affect astrocytic function still remains unknown. Taken together, these studies suggest that *C9orf72* haploinsufficiency, resulting from the C9-HRE, may lead to p62 accumulation, which could also propagate to other cells (Madill et al., 2017). Additionally, changes in NFκB and EDN1 signaling, disturbed glutamate metabolism, and lysosomal alterations might be phenotypic features of C9-HRE astrocytes with potentially altered physiological functions (Figure 1).

## Microglia in C9-FTLD and C9-ALS Models

### Features of C9-HRE-Expressing Microglia

Depending on environmental cues, microglia can switch from resting to activated phenotype, characterized by elevated expression of CD68 (Choi et al., 2017; Hendrickx et al., 2017). Studies on *post-mortem* brains have shown increased microglial activation based on cell morphology and Iba1 and CD68 immunoreactivity in C9-FTLD and C9-ALS *vs*. sporadic FTLD and ALS cases. Significantly increased microglial activation according to number and morphology (ramified *vs*. ameoboid) of CD68-positive cells in frontal and temporal gray and white matter was detected in FTLD cases compared to controls, although there were no differences between C9-FTLD and sporadic FTLD cases (Lant et al., 2014). Also, augmented microglial activation based on CD68 immunoreactivity and cell morphology in the white matter of medulla and motor cortex of C9-ALS patients compared to non-C9-ALS cases has been reported. Iba1 immunoreactivity was also increased, indicating potentially increased number of microglia (Brettschneider et al., 2012). Higher CD68 immunoreactivity in the white matter of motor cortex, medulla, mid-crus cerebri, and lateral and anterior corticospinal tracts of C9-ALS patients (Cooper-Knock et al., 2012) and in the body and genu of corpus callosum of C9-HRE-carrying vs. non-carrying ALS patients has been detected (Cardenas et al., 2017), suggesting that microglial activation and infiltration in the brain might take place at least in late stages of C9-FTLD and C9-ALS. It would be crucial to assess at different stages of the disease process whether the phenotype of C9-HRE-carrying activated microglia is pro- or anti-inflammatory.

Iba1-positive microglia of C9-ALS patient *post-mortem* motor cortex and spinal cord contained enlarged lysosomes based on LAMP1 immunoreactivity compared to sporadic ALS cases (O'Rourke et al., 2016), suggesting lysosomal alterations in C9-HRE carriers. Total LAMP1 levels were not significantly changed in the frontal cortex of C9-ALS cases compared to sporadic ALS nor between FTLD and ALS and control samples (Umoh et al., 2018). This might suggest that (i) altered lysosomal morphology does not correlate with total LAMP1 levels or (ii) that microglia from spinal cord and frontal cortex show different lysosomal features. Enlarged microglial lysosomes may emerge under different conditions, such as cathepsin B and L inhibition and subsequent prevention of autophagosome-lysosome fusion (Jung et al., 2015), defects in lysosomal fission (Durchfort et al., 2012), progranulin deficiency (Evers et al., 2017) or TMEM106b overexpression (Nicholson and Rademakers, 2016), resulting in decreased lysosomal degradation capacity (Jung et al., 2015; Nicholson and Rademakers, 2016), and upon phagocytosis of fibrillar amyloid β, resulting in cathepsin B release (Halle et al., 2008). Further investigations are warranted related to the number of microglia with enlarged lysosomes and factors triggering such a phenotype, as well as understanding whether the enlarged lysosomes reflect dysfunction or improved function. Nevertheless, enhanced Iba1 and CD68 immunoreactivity and enlarged lysosomes may be considered as typical features of late stage C9-FTLD or C9-ALS microglia (Figure 2).

### Neuron-Microglia Crosstalk Might Contribute to C9-HRE Microglia Phenotype

Expression of pathological C9-HRE in mouse cortex has been shown to upregulate *Iba1* expression, but it is unclear whether this is due to cell-autonomous or non-cell autonomous effects (Nicholson et al., 2018). Significantly enhanced CD68 and Iba1 expression was detected at 6 months of age in the spinal cord of transgenic mice expressing poly-GA_149_ specifically in neurons. Increased microglial activation was absent in brain areas where the neurons did not exhibit poly-GA pathology. At this time point, no significant neuronal loss could be detected. However, the mice demonstrated enhanced TDP-43 phosphorylation, but no translocation or inclusions, and mild behavioral deficits, indicating that microglial activation might precede severe neuronal dysfunction. Interestingly, 1 month-old mice did not exhibit elevated Iba1 but already elevated CD68 expression (Schludi et al., 2017), implicating that enhanced CD68 expression in microglia may precede increased Iba1 expression. In contrast, expression of poly-GA_50_ did not increase Iba1 levels or cause TDP-43 pathology at 6 months of age, but the mice showed behavioral impairments and neurodegeneration (Zhang et al., 2016). Expression of poly-GR_100_ led to elevated Iba1 levels in mouse cortex and hippocampus, which peaked at 1.5 months of age. Notably, at this time, neuronal loss and brain atrophy, but no TDP-43 pathology, were already detectable in hippocampus and cortex (Zhang et al., 2018). These studies suggest that DRP length or type and/or concomitant additional factors, such as TDP-43 phosphorylation, might differentially regulate microglial Iba1 levels and activation. Also, neuron-microglia crosstalk might contribute to the activation of microglia.

### Decreased C9orf72 Levels May Influence C9-HRE Microglia Phenotype

Human *C9orf72* and its mouse ortholog 3110043O21Rik are strongly expressed in myeloid cells, including microglia (O'Rourke et al., 2016; Rizzu et al., 2016; Iyer et al., 2018). *C9orf72*^−/−^ mice show severe autoimmune phenotypes, elevated levels of inflammatory cytokines [IL-12, IL-10, tumor necrosis factor (TNF) α, IL-17] and monocyte chemoattractant protein 1 in serum and pro-inflammatory macrophage polarization (Atanasio et al., 2016; O'Rourke et al., 2016; Sullivan et al., 2016). Microglia of *C9orf72*^−/−^ mice showed phenotypes ranging from accumulation of enlarged lysosomes and enhanced LAMP1 immunoreactivity, strongly increased expression of IL-6 and IL-1β under basal conditions (O'Rourke et al., 2016) to no changes in LAMP1 or cathepsin D immunoreactivity (Sullivan et al., 2016). However, microglia from hemizygous mice were not reported to show significant increases in pro-inflammatory cytokine levels or lysosomal changes (O'Rourke et al., 2016). Furthermore, even in *C9orf72*^−/−^ mice, no increase in Iba1 immunoreactivity or changes in the morphology, number, or distribution of microglia were detected in contrast to human *post-mortem* brain as described above (Koppers et al., 2015; Sullivan et al., 2016). On the other hand, antisense oligonucleotides targeting *C9orf72* transcripts induced the mRNA levels of *triggering receptor expressed on myeloid cells* (*TREM*) *2, tyrosine kinase binding protein* (*TYROBP*), *C1q B chain* (*C1qb*) and *C3a receptor 1* (*C3ar1*), all predominantly expressed in microglia and regulating central microglial functions, including activation, pruning, and phagocytosis (Lagier-Tourenne et al., 2013), suggesting that reduced *C9orf72* levels associate with alterations in these microglial functions. Altogether, the current data suggest that *C9orf72* haploinsufficiency might not underlie the C9-ALS and C9-FTLD microglial phenotypes as assessed by Iba1 and CD68 immunoreactivity. Whether lysosomal changes resulting from reduced *C9orf72* levels occur in microglia needs further investigation. *C9orf72* knockout in HEK293T cells leads to enlarged lysosomes, suggesting that similar effects might also occur in other cell types (Amick et al., 2016). However, enhanced *TREM2, TYROBP, C1qb*, and *C3ar1* expression might represent additional phenotypic features of C9-HRE microglia (Figure 2). Interestingly, also progranulin-deficient mice show enlarged lysosomes, increased *CD68, TYROBP, TREM*2 and complement factor expression (Evers et al., 2017) and Iba1 immunoreactivity (Tanaka et al., 2014).

## Conclusions and Future Perspectives

Here, we have discussed the potential phenotypes of C9-HRE astrocytes and microglia based on the current literature. In general, FTLD and ALS are complex multifactorial diseases involving multi-cellular components. Thus, dysfunction of one cell type only might not be enough to trigger neurodegeneration but different cell types and their crosstalk are likely to contribute. Regarding C9-HRE-associated FTLD and ALS, astrocytes are suggested to mediate neurotoxic effects, but so far there is no conclusive experimental evidence of direct contribution of microglia to neurodegeneration in C9-FTLD and C9-ALS models. The reason for this might be that the current approaches have focused mostly on *C9orf72* haploinsufficiency when modeling the C9-HRE-related effects in microglia. These studies have suggested that *C9orf72* depletion, which leads to severe dysfunction of the immune system, might affect macrophages and microglia differently. Since neurons with C9-HRE-associated pathological features rather than *C9orf72* haploinsufficiency might cause microglia activation, concomitant modeling of the loss-of-function and gain-of-toxic function mechanisms might help to decipher whether and how microglia may take part in C9-FTLD and C9-ALS pathogenesis. Also, since loss of *C9orf72* has been linked to autoimmune phenotypes, it would be important to also investigate the crosstalk between the peripheral immune system and CNS-resident cells in the future. While the C9-HRE microglial phenotypes might result, at least partially, from neuron-microglia crosstalk, astrocytic neurotoxicity appears to derive from intrinsic factors, which then display non-cell autonomous deleterious effects on neurons. Since C9-HRE astrocytes show defects in EV-based communication with neurons, investigating whether the crosstalk between astrocytes and microglia of C9-HRE carriers is changed as well, would reveal further insights into mechanisms potentially underlying C9-HRE-associated ALS and FTLD. Future investigations are warranted to uncover potential spatial and temporal contributions of glia to onset and progression of C9-FTLD and C9-ALS. Also, even though C9-FTLD and C9-ALS share overlapping genetic background and pathological features, it will be interesting to find out the similarities and potential differences in the effects of microglia and astrocytes on the pathogenesis of these two diseases. As the presently available literature on glia in the context of C9-HRE is still limited, we hope that this summary of the current knowledge and the hypotheses presented will help to design future experiments for deciphering the so far poorly understood role of astrocytes and microglia in disease pathogenesis and for identifying potential novel biomarker and/or therapeutic target candidates for FTLD and ALS.

## Author Contributions

HR and AH outlined and drafted the manuscript. HR, SL, NH, KK, AC, ES, MM, TN, AR, MH, and AH participated in the writing and editing the manuscript. All authors have accepted the contents of the submitted manuscript.

### Conflict of Interest Statement

The authors declare that the research was conducted in the absence of any commercial or financial relationships that could be construed as a potential conflict of interest.
